# Methoden, mit dem Rauchen aufzuhören – eine quantitative Online-Befragung zu förderlichen und hinderlichen Faktoren (RauS-Studie)

**DOI:** 10.1007/s00103-024-03970-x

**Published:** 2024-11-07

**Authors:** Bernd Werse, Larissa Steimle, Heino Stöver

**Affiliations:** Institut für Suchtforschung (ISFF), FB4: Soziale Arbeit und Gesundheit, Frankfurt University of Applied Sciences, Nibelungenplatz 1, 60318 Frankfurt am Main, Deutschland

**Keywords:** Rauchen, Rauchstopp, Tabak, Hindernisse, Schadensminderung, Smoking, Smoking cessation, Tobacco, Barriers, Tobacco harm reduction

## Abstract

**Hintergrund:**

Rauchen ist nach wie vor das größte vermeidbare Gesundheitsrisiko in Deutschland, weshalb eine höhere Erfolgsquote bei der Rauchentwöhnung der Gesundheit aller zugutekommen würde. Um dies zu erreichen, ist eine bessere Kenntnis über Rauchstoppmethoden sowie förderliche und hinderliche Faktoren beim Rauchstopp erforderlich. Daher sollte mit der RauS-Studie untersucht werden, mit welchen Methoden Rauchstoppversuche unternommen wurden, wie hilfreich diese Methoden waren und welche Faktoren dabei als förderlich bzw. hinderlich empfunden wurden.

**Methoden:**

Von März bis Dezember 2022 wurde eine quantitative Online-Befragung unter mindestens 14-jährigen aktuellen oder ehemaligen Raucher*innen (*N* = 6192) durchgeführt.

**Ergebnisse:**

93 % der Stichprobe haben mindestens einmal versucht, mit dem Rauchen aufzuhören. Von den Befragten, die keine E‑Zigaretten/Tabakerhitzer nutz(t)en, ist die „eigene Willenskraft“ die am häufigsten genutzte und als am hilfreichsten wahrgenommene Methode. Von Personen, die E‑Zigaretten/Tabakerhitzer für einen Rauchstopp verwendet haben, werden E‑Zigaretten mit Nikotin am häufigsten genutzt und als am hilfreichsten beurteilt. Gesundheitliche Folgen werden als motivationsfördernd empfunden. An einem Rauchstopp hindern vor allem mit dem Rauchen assoziierte Rituale, während Entzugssymptome eine deutlich geringere Rolle spielen.

**Diskussion:**

Insgesamt zeigen die Ergebnisse, dass die Fokussierung auf „Nikotinabhängigkeit“ bei der Rauchentwöhnung überdacht und soziale und rituelle Dimensionen stärker angesprochen werden sollten. E‑Zigaretten stellen zumindest für einen Teil der Betroffenen eine durchaus hilfreiche Methode zur Schadensminderung und/oder zum Rauchstopp dar.

## Abstract (en)

**Background:**

Smoking is still the largest preventable health risk in Germany, which is why a higher success rate in smoking cessation would benefit both public and individual health. In order to achieve this, a better understanding of the smoking cessation methods used and the factors that facilitate and prevent smoking cessation is required. The RauS study therefore aimed to investigate which methods have been used in attempts to stop smoking, how successful these methods have been, and which factors have been perceived as helping or hindering smoking cessation.

**Methods:**

From March to December 2022 a quantitative online survey was conducted among current or former smokers over 14 years of age (*N* = 6192).

**Results:**

Of the sample, 93% have tried to quit smoking at least once. In general, for the respondents who did or did not use e‑cigarettes/tobacco heaters, “personal willpower” was the most frequently used and perceived as the most helpful method. Among people who have used e‑cigarettes/tobacco heaters to quit smoking, e‑cigarettes with nicotine were used most frequently and were rated as the most helpful. Potential or concrete health consequences were most likely to be perceived as motivating. Rituals associated with smoking were the main barrier to quit smoking, while withdrawal symptoms played a much smaller role.

**Discussion:**

Overall, the results show that the focus on “nicotine addiction” in smoking cessation should be reconsidered, and social and ritual dimensions should be addressed more strongly. For at least some of those affected, e‑cigarettes may be a helpful method of reducing harm and/or quitting smoking.

## Hintergrund

Deutschland gilt weiterhin als ein Tabakhochkonsumland. Laut dem Deutschen Krebsforschungszentrum sterben in Deutschland jährlich über 127.000 Menschen vorzeitig an den Folgen des Tabakkonsums. Zu diesen weitreichenden gesundheitlichen Folgen des Tabakkonsums kommen gesamtgesellschaftliche Kosten von jährlich 97 Mrd. € sowie erhebliche ökologische Schäden [1].

Vor diesem Hintergrund stellt die Unterstützung von rauchausstiegswilligen Menschen bzw. die generelle Förderung von Motivationen zum Rauchausstieg eine große gesundheitspolitische Herausforderung dar. Wenn im Folgenden von „Rauchstopp“, „Rauchstoppversuchen“ oder „Nichtraucher*innen“ gesprochen wird, ist die Abstinenz von Produkten gemeint, bei denen Tabak verbrannt wird. Dazu gehört allen voran die Zigarette, aber auch z. B. Zigarren oder Pfeifen. Zur Unterstützung eines Rauchstoppversuchs werden in Deutschland evidenzbasierte Methoden entsprechend der S3-Leitlinie [2] empfohlen. Darunter fallen Kurzberatung durch Mediziner*innen oder Apotheker*innen, verhaltenstherapeutische Einzel- oder Gruppentherapien, Nikotinersatztherapien (Pflaster, Kaugummis o. Ä.) sowie die Medikamente Vareniclin und Bupropion. Diese Methoden erzielen zwar unter kontrollierten Bedingungen vergleichsweise gute Ergebnisse [3], werden aber nur von wenigen Raucher*innen genutzt [4]. So wurde in der repräsentativen DEBRA-Studie (Deutsche Befragung zum Rauchverhalten) deutlich am häufigsten, von fast 60 % der relevanten Befragten, die „eigene Willenskraft“ als Methode zum Rauchstopp verwendet [4], was sich auch in anderen repräsentativen Studien im In- und Ausland bestätigt [5, 6].

Insgesamt zeigen Studien, dass die Bereitschaft zum Aufhören unter aktuell Rauchenden grundsätzlich hoch ist. Trotzdem scheitern viele Rauchstoppversuche bzw. eher wenige Raucher*innen unternehmen ernsthafte Versuche, mit dem Rauchen aufzuhören [4]. Neben den genannten Studien gibt es in Deutschland wenig Forschung über Rauchstoppmethoden. Insbesondere Faktoren, die Rauchstoppversuche begünstigen oder erschweren, waren bislang im deutschsprachigen Raum in keiner Studie mit nennenswerter Stichprobe ein Thema. Im internationalen Raum hingegen gibt es durchaus Studien, die sich mit diesem Thema auseinandersetzen und beispielsweise untersuchen, wie sich Atemwegs- und Herz-Kreislauf-Erkrankungen auf die Rauchentwöhnungsrate auswirken, mit dem Ergebnis, dass weder Atemwegserkrankungen noch kardiovaskuläre Risikofaktoren signifikant zu einem Rauchstopp motivieren, wobei eine akute Episode einer koronaren Herzkrankheit die Rauchentwöhnung fördert [7]. Weiterhin gibt es Studien die zeigen, dass einzelne Rauchstoppmethoden, wie beispielsweise Nikotin-E-Zigaretten, Rauchstoppversuche unterstützen können [8]. Darüber hinaus gibt es Untersuchungen zu hinderlichen und förderlichen Faktoren in bestimmten Kontexten, wie beispielsweise der ärztlichen Erstversorgung, in denen als Haupthindernis identifiziert wurde, dass sowohl Raucher*innen als auch medizinisches Fachpersonal häufig darauf warten, dass die jeweils andere Partei eine Initiative zum Start eines Rauchstoppversuchs startet [9].

Derartige Studienergebnisse können indes nicht zwangsläufig auf Deutschland übertragen werden; zudem verweisen sie nur auf einzelne förderliche und hinderliche Faktoren. Solche Faktoren sowie die Beurteilung von Rauchstoppmethoden als hilfreich oder weniger hilfreich stehen in einem engen Zusammenhang mit der Tabakkontrollpolitik [7], die sich länderspezifisch sehr stark unterscheidet. Ein Beispiel sind E‑Zigaretten: Auch wenn Langzeitstudien fehlen, gibt es mittlerweile hinreichende Evidenz dafür, dass E‑Zigaretten deutlich weniger schädlich sind als Verbrennungszigaretten und Rauchstoppversuche wirksam unterstützen können [8, 10, 11]. Aus diesem Grund werden E‑Zigaretten beispielsweise im Vereinigten Königreich aktiv im Rahmen einer gesundheitspolitischen Strategie empfohlen [12]. In Deutschland ist dies nicht der Fall und es herrscht Verunsicherung in der Bevölkerung darüber, ob solche Produkte schädlicher oder weniger schädlich sind als Verbrennungszigaretten [13]. Es kann davon ausgegangen werden, dass sich diese Verunsicherung auch darauf auswirkt, ob diese Methode als hilfreich oder weniger hilfreich empfunden wird [14].

Dementsprechend fehlt es in Deutschland an Studien zur näheren Ergründung von angewendeten Rauchstoppmethoden. Mit der vorliegenden Studie sollten insbesondere förderliche und hinderliche Faktoren in den Blick genommen werden. Dabei stand die Beantwortung folgender Fragen im Mittelpunkt:

1. Welche Methoden wurden zum Rauchstoppversuch genutzt und als wie hilfreich wurden diese empfunden?
2. Welche hinderlichen und förderlichen Faktoren identifizieren die Befragten im Hinblick auf ihre Rauchstoppversuche?

Eine erste Übersicht über die Ergebnisse dieser Studie wurde von Bernd Werse et al. im von Heino Stöver herausgegebenen Sammelband *Die Zigarette liegt in den letzten Zügen* veröffentlicht [15].

## Methoden

Zur Beantwortung der Forschungsfragen wurde von März bis Dezember 2022 eine Online-Befragung unter dem Titel „RauS“ (Rauchstopp-Studie) durchgeführt. Der Fragebogen richtete sich an alle mindestens 14-Jährigen, die der deutschen Sprache mächtig sind und:

1. entweder aktuell Zigaretten, Zigarren, Pfeifen oder Ähnliches rauchen oder
2. dies in der Vergangenheit über mindestens 12 Monate hinweg mindestens wöchentlich getan haben.

Neben soziodemografischen Daten wurden Prävalenzraten unterschiedlicher Produkte sowie die Intensität von Abhängigkeit und Rauchstoppmethoden abgefragt, die teilweise aus der bereits genannten DEBRA-Studie übernommen wurden. Schwerpunkt der Erhebung waren Fragen zur Nutzung und Bewertung von Methoden zum Rauchstopp. Neben Antworten in Kategorien gab es bei diversen Fragen die Möglichkeit offener Texteingaben. Vor der Erhebung wurde ein explorativer Pretest in Form eines Kurzfragebogens mit offenen Antwortfeldern durchgeführt, auf dem u. a. viele der Antwortkategorien basieren (*n* = 25).

Zur Verbreitung des Fragebogens wurden diverse Social-Media-Accounts (Twitter, Facebook, Instagram) genutzt. Zusätzlich wurden Organisationen wie die Deutsche Hauptstelle für Suchtfragen (DHS) und die Bundeszentrale für gesundheitliche Aufklärung (BZgA) sowie Wissenschaftler*innen, Journalist*innen und andere Multiplikator*innen um Weiterverbreitung gebeten. Weiterhin wurde der Link über Pressemitteilungen der Frankfurt University of Applied Sciences (FRA-UAS) und der Goethe-Universität bekanntgegeben und auf den o. g. Social-Media-Plattformen sowie in Handyspielen Werbung geschaltet. Ziel war es, eine möglichst große Stichprobe zu erreichen, wobei nicht beabsichtigt war, Repräsentativität zu erzielen. Unter den Teilnehmenden, die eine E‑Mail-Adresse angaben, wurden 10 Universalgutscheine à 25 € verlost.

### Datenanalyse

Der Fragebogen enthielt überwiegend ankreuzbare Antwortkategorien bzw. Felder für Zahleneingaben, wobei an einigen Stellen auch Freitextfelder genutzt wurden. Entsprechend wurden die Fragen mit der statistischen Analysesoftware SPSS 22 mit deskriptiver Statistik, Gruppen- und Mittelwertvergleichen (X^2^, ANOVA) in einzelnen Fällen auch Pearson-Korrelationen zwischen Variablen sowie Risikoschätzern (Odds Ratio/OR) ausgewertet. Statistische Vergleiche im Ergebnisteil enthalten den jeweiligen Koeffizienten sowie das Signifikanzniveau (*p* < 0,05, *p* < 0,01, *p* < 0,001 sowie n. s. für nichtsignifikante Differenzen).

Für alle im Folgenden präsentierten Analysen wurden auch Geschlechtervergleiche durchgeführt. Häufig zeigten sich keine signifikanten Differenzen zwischen den Geschlechtern. Die Unterschiede, die statistische Signifikanz erreichen, sind im Text angeführt.

## Ergebnisse

Der Fragebogen wurde insgesamt 8752-mal zumindest teilweise ausgefüllt. Bei 7307 dieser Fragebögen wurden mindestens 80 % der Fragen beantwortet. Von diesen 7307 Fragebögen wurden diejenigen abgezogen, bei denen zentrale Fragen zum Rauchstopp gefehlt hatten oder die von Personen ausgefüllt wurden, die nie oder nicht länger als 1 Jahr mindestens wöchentlich geraucht haben. Nach Abzug dieser Personen wurden insgesamt 6192 Fragebögen in die Auswertung eingeschlossen. 3 % haben mehr als 10 % der Fragen nicht beantwortet, weitere 23 % zwischen 6 % und 10 % (Median: 4 %).

### Stichprobe

Nach einer kritischen Analyse der Stichprobe zeigte sich im Vergleich zu anderen Studien [16], dass aufgrund der Erwähnung der Studie in Videos von 2 Dampfer-Influencern offenbar überproportional viele E‑Zigaretten-Konsument*innen in der Stichprobe vertreten sind. Um diesem Bias entgegenzuwirken, wurde die Gesamtstichprobe (*N* = 6192) in 2 Gruppen aufgeteilt:

1. *mit E:* Personen, die jemals E‑Zigaretten (mit oder ohne Nikotin)^1^ oder Tabakerhitzer^2^ für einen Rauchstoppversuch genutzt haben (*n*_*1*_ = 3690);
2. *ohne E:* Personen, die *nie *E‑Zigaretten oder Tabakerhitzer für einen Rauchstoppversuch genutzt haben (*n*_*2*_ = 2502).

Bei der ersten Gruppe (1) sind dementsprechend nur Personen enthalten, die mindestens einen Rauchstoppversuch unternommen haben, während in Gruppe (2) auch Menschen enthalten sind, die noch nie versucht haben, mit dem Rauchen aufzuhören. Gruppe 2 rekrutiert sich vermutlich zu einem großen Teil aus Personen, die über Online-Werbung erreicht wurden. Hiermit wurde ein Querschnitt zumindest der online-affinen, raucherfahrenen Bevölkerung angesprochen.

73 % der Gesamtstichprobe (*N* = 6192) ordnen sich der Kategorie männlich, 27 % weiblich und 0,3 % der Kategorie divers zu. Während der Männeranteil der Gesamtstichprobe im Vergleich zu anderen Studien [16] hoch erscheint, liegt er in der Gruppe *ohne E* mit 58 % sehr nahe am Wert, der in anderen Studien zu aktuell Rauchenden genannt wird. In der Gruppe *mit E* ist der Männeranteil mit 82 % deutlich höher, was in der Tendenz mit Erkenntnissen aus Repräsentativerhebungen einhergeht, die zeigen, dass Männer beim Konsum von E‑Zigaretten deutlich überrepräsentiert sind [17].

Das Durchschnittsalter (*n* = 5706) liegt mit 45,1 Jahren (Standardabweichung [SD] 12,6; Median 45 Jahre) sehr nahe am Durchschnittsalter der deutschen Gesamtbevölkerung [18]. Das Haushaltsnettoeinkommen der Stichprobe (erhoben in 5 Kategorien) liegt eher unter dem der deutschen Allgemeinbevölkerung [19], der Bildungsgrad hingegen etwas darüber ([20]; Tab. 1). Neben den 40 % mit Abitur o. Ä. haben 35 % einen Real-, 17 % einen Hauptschulabschluss, 1 % keinen und 7 % einen sonstigen Abschluss.

**Tab. 1:** Einige Basisdaten im Gruppenvergleich

	Mit E	Ohne E	Gesamt	*N*
Geschlecht: männlich	82 % (*n* = 2940)	58 % (*n* = 1221)	73 % (*n* = 4161)	5709
Durchschnittsalter	44 J	47 J	45,1 J	5706
Haushalts-Nettoeinkommen über 2500 €	52 % (*n* = 1845)	45 % (*n* = 915)	49 % (*n* = 2760)	5581
Schulabschluss Abitur oder Ähnliches	37 % (*n* = 1338)	44 % (*n* = 925)	40 % (*n* = 2263)	5681
Aktuelle Raucher*innen	20 % (*n* = 735)	50 % (*n* = 1234)	32 % (*n* = 1969)	6174

Die Tabelle wurden in Teilen aus [15] übernommen, mit freundlicher Genehmigung © Schulz-Kirchner Verlag GmbH 2024

32 % der Gesamtstichprobe (*N* = 6192) rauchen Tabakprodukte. Weitere 7 % rauchen nicht, konsumieren aber zumindest gelegentlich Cannabis, wobei unklar ist, wie viele davon ggf. Cannabis mit Tabak (z. B. im Joint) rauchen. 48 % konsumieren zum Zeitpunkt der Befragung ausschließlich E‑Zigaretten und 13 % geben an, derzeit kein inhalierbares Produkt zu nutzen.

### Rauchstopp- und Reduktionsversuche

Unter den aktuellen Raucher*innen (*n* = 1949) geben 26 % an, dass sie (eigentlich) nicht aufhören wollen, und 15 %, dass sie noch nicht darüber nachgedacht haben, wann sie aufhören wollen. 59 % wollen bald aufhören.

Von denjenigen, die nicht aufhören wollen zu rauchen (26 %), geben 39 % an, den Konsum etwas (z. B. weniger pro Tag), und 29 %, den Konsum deutlich (z. B. auf besondere Gelegenheiten) reduzieren zu wollen. 32 % geben an, ihren Konsum nicht reduzieren zu wollen.

93 % der Gesamtstichprobe (*N* = 6192) haben mindestens einmal im Leben einen Rauchstoppversuch unternommen. Das sind 89 % der aktuellen Raucher*innen (*n* = 1981) und 94 % derer, die bereits aufgehört haben (*n* = 4211). Aus der Gruppe *mit E* haben per Definition 100 %, aus der Gruppe *ohne E* 82 % mindestens einmal versucht, mit dem Rauchen aufzuhören.

Die Befragten aus der Gesamtstichprobe haben im Schnitt 6,4 Rauchstoppversuche^3^ unternommen (*mit E*: 6,6; *ohne E*: 5,8), davon 3,8 „ernsthafte“ Versuche^4^ (*mit E*: 3,8; *ohne E*: 3,6).

Gefragt danach, ob sie schon einmal versucht haben, ihren Konsum zu reduzieren,^5^ gaben 39 % der Gesamtstichprobe an, dies bisher nicht versucht zu haben. 46 % haben bisher mindestens einmal versucht, ihren Konsum zu reduzieren. Hinzu kommen 15 %, die ihren Konsum unbewusst^6^ reduziert haben. 52 % waren mit der Reduktion vorübergehend erfolgreich, 10 % haben dauerhaft ihren Konsum reduziert und 14 % später ganz aufgehört. Dauerhaft Erfolgreiche haben dafür im Schnitt etwa 5 Reduktionsversuche gebraucht, wobei gänzlich Erfolglose fast doppelt so viele Versuche hinter sich haben.

Bei Frauen zeigen sich signifikant höhere Werte für Reduktionsversuche (bewusst: 53 %, nicht bewusst: 16 %) als bei Männern (bewusst: 43 %, nicht bewusst: 15 %; Χ^2^(4, *n* = 5700) = 56,6; *p* < 0,001), wobei Männer mit Reduktionsversuchen etwas häufiger infolgedessen mit dem Rauchen aufgehört haben als Frauen (14 % vs. 12 %). Letztere nennen etwas häufiger eine nur vorübergehende Reduktion ihres Konsums (56 % vs. 49 %; Χ^2^(6, *n* = 3473) = 15,7; *p* < 0,05).

### Maßnahmen zum Rauchstopp

Bei der Gruppe *ohne E *(*n*_*2*_ = 2502) zeigt sich, dass die mit Abstand am häufigsten verwendete Maßnahme zum Rauchstopp mit 69 % die „eigene Willenskraft“ darstellt.^7^ Weit dahinter folgt „Buch“ mit 25 % und auf Platz 3 mit 22 % „kontrolliertes Rauchen“^8^. An vierter Stelle liegen mit 20 % Nikotinersatztherapien, die von den evidenzbasierten Methoden somit am häufigsten genutzt wurden (Tab. 2).

**Tab. 2:** Jemals verwendete Maßnahmen für Rauchstoppversuche im Gruppenvergleich (%)

	Mit E (*n* = 3690)	Ohne E (*n* = 1794)	Gesamt (*n* = 5484)	95 %-KI (Gesamt)
*Evidenzbasierte Methoden laut S3-Leitlinie (DG-Sucht 2021)* ^*a*^				
Kurzberatung Arzt/Ärztin/Apotheker*in	7	4	6	5–7
Verhaltenstherapeut. Behandlung od. telefonische Beratung	5	5	5	4–5
Nikotinersatztherapie	30	20	27	25–28
Medikament zur Rauchentwöhnung	5	5	5	4–6
*Weitere Methoden*				
E‑Zigaretten mit Nikotin	94	– ^b^	64	62–65
E‑Zigaretten ohne Nikotin	28	– ^b^	19	18–20
App oder Website	3	6	4	3–4
Buch	23	25	24	23–25
Hypnotherapie	4	6	5	4–5
Akupunktur	8	8	8	7–8
Heilpraktiker*in	3	4	3	3
Eigene Willenskraft	57	69	61	60–62
Unterstützung soziales Umfeld	16	16	16	15–17
Tabakerhitzer	8	– ^b^	6	5–6
Nikotin-Pouches/Snus	5	2	4	4–5
Kräuterzigaretten	6	6	6	6–7
Cannabis	10	13	11	10–12
Kontrolliertes Rauchen	34	22	30	29–31
Beschränkung auf seltene Gelegenheiten	22	19	21	20–22
Ortswechsel	6	9	7	6–8
Ersatzrituale	5	10	7	6–8
Sonstige	2	13	6	5–6

Die Tabelle wurden in Teilen aus [15] übernommen, mit freundlicher Genehmigung © Schulz-Kirchner Verlag GmbH 2024

^a^ S3-Leitlinie der Deutschen Gesellschaft für Suchtforschung und Suchttherapie e. V. 2021

^b^ Per definitionem konnten E‑Zigaretten und Tabakerhitzer nicht von den Teilnehmer*innen aus der Gruppe ohne E genannt werden

Bei der Gruppe *mit E* (*n*_*1*_ = 3690) ist die mit Abstand am häufigsten verwendete Maßnahme zur Unterstützung eines Rauchstoppversuchs die „E-Zigarette mit Nikotin“ (94 %), gefolgt von der „eigenen Willenskraft“ (57 %). Auf Platz 3 liegt „kontrolliertes Rauchen“ (34 %) und ebenfalls auf Platz 4 liegen mit 30 % die Nikotinersatztherapien.

Frauen nutzen etwas (signifikant) häufiger 3 der evidenzbasierten Methoden („verhaltenstherapeutische Behandlung“, „Medikamente zur Rauchentwöhnung“ und „Nikotinersatztherapie“). Darüber hinaus nennen Frauen eher Erfahrungen mit verhaltensbezogenen Methoden (z. B. „Ersatzrituale“) sowie alternativmedizinische Ansätze (z. B. „Hypnotherapie“) und „sonstige Methoden“. Der größte Geschlechterunterschied zeigt sich bei der Kategorie „Buch“, die von 33 % der Frauen und 20 % der Männer (Χ^2^ (2, *n* = 5265) = 100,1; *p* < 0,001; OR (*n* = 5251) = 1,97^9^) ausprobiert wurde. Männer nutzen hingegen – folgerichtig angesichts des überproportional großen Männeranteils in der Gruppe „mit E“ – weitaus häufiger „E-Zigaretten mit Nikotin“ als Frauen (73 % vs. 40 %; Χ^2^(2, *n* = 5265) = 514,6; *p* < 0,001; OR (*n* = 5251) = 0,24), daneben auch „E-Zigaretten ohne Nikotin“, „Nikotin-Pouches/Snus“ und „Cannabis“.

Wie hilfreich die einzelnen Rauchstoppmethoden empfunden wurde, beurteilten jene Befragten, welche die jeweilige Methode ausprobiert haben, auf einer 5‑teiligen Skala (Tab. 3).^10^ Befragte *ohne E* empfinden die „eigene Willenskraft“ (4,2) als hilfreichste Methode, gefolgt von „sonstige Methoden“ (4,0). Bei der Kategorie „sonstige Methoden“ werden am häufigsten Krankheiten, Verletzungen und Operationen genannt, gefolgt von Schwangerschaft bzw. Geburt (von Frauen und Männern), wobei sich ansonsten ein extrem breites Spektrum an Angaben zeigt.

**Tab. 3:** Bewertung, inwiefern bestimmte Rauchstoppmaßnahmen hilfreich gewesen sind, entsprechend einer 5–teiligen Skala (1 = überhaupt nicht hilfreich, 5 = sehr hilfreich); Mittelwerte (Standardabweichungen) bezogen auf diejenigen, welche die jeweilige Maßnahme ausprobiert haben, im Gruppenvergleich

	Mit E	Ohne E	Gesamt
Eigene Willenskraft (*n* = 3311)	3,1 (1,2)	4,2 (1,1)	3,5 (1,3)
Sonstige (*n* = 297)	2,8 (1,4)	4,0 (1,4)	3,6 (1,5)
Cannabis (*n* = 334)	3,3 (1,3)	3,9 (1,1)	3,5 (1,3)
Unterstützung soz. Umfeld (*n* = 893)	2,9 (1,1)	3,7 (1,1)	3,1 (1,2)
Ersatzrituale (*n* = 375)	2,6 (1,1)	3,7 (1,2)	3,2 (1,3)
Ortswechsel (*n* = 372)	2,7 (1,2)	3,7 (1,2)	3,1 (1,3)
App oder Website (*n* = 209)	3,0 (1,2)	3,6 (1,2)	3,3 (1,2)
Buch (*n* = 1279)	2,4 (1,1)	3,3 (1,3)	2,7 (1,3)
Verhaltenstherapeutische Behandlung (*n* = 254)	2,1 (1,1)	3,2 (1,2)	2,5 (1,2)
Medikament zur Rauchentwöhnung (*n* = 268)	2,2 (1,2)	3,1 (1,4)	2,5 (1,3)
Nikotin-Pouches/Snus (*n* = 229)	2,5 (1,2)	2,9 (1,5)	2,5 (1,3)
Hypnotherapie (*n* = 246)	2,2 (1,2)	2,9 (1,5)	2,4 (1,4)
Heilpraktiker (*n* = 158)	1,9 (1)	2,8 (1,2)	2,3 (1,1)
Nikotinersatztherapie (*n* = 1445)	1,7 (0,9)	2,8 (1,3)	2,0 (1,1)
Akupunktur (*n* = 417)	1,9 (1)	2,7 (1,3)	2,2 (1,2)
Kurzberatung Arzt/Ärztin/Apotheker*in (*n* = 328)	1,9 (0,9)	2,7 (1,2)	2,0 (1)
Kontrolliertes Rauchen (*n* = 1634)	2,1 (1)	2,6 (1,2)	2,2 (1,1)
Beschränkung auf seltene Gelegenheiten (*n* = 1156)	1,9 0,9)	2,6 (1,2)	2,1 (1,1)
Kräuterzigaretten (*n* = 337)	1,7 (0,9)	2,5 (1,2)	2,0 (1,1)
E‑Zigaretten mit Nikotin (*n* = 3455)	4,7 (0,8)	–	4,7 (0,8)
E‑Zigaretten ohne Nikotin (*n* = 1026)	3,5 (1,2)	–	3,5 (1,2)
Tabakerhitzer (*n* = 302)	2,8 (1,2)	–	2,8 (1,2)

Die Tabelle wurden in Teilen aus [15] übernommen, mit freundlicher Genehmigung © Schulz-Kirchner Verlag GmbH 2024

Bei den Befragten *mit E* wird die E‑Zigarette mit Nikotin (4,7) mit Abstand als hilfreichste Methode angesehen, gefolgt von „E-Zigaretten ohne Nikotin“ (3,5; Tab. 3).

Werden die 2 Fragen nach den genutzten Methoden und der als besonders hilfreich empfundenen Maßnahme zusammengedacht, ergibt sich für die Gruppe *ohne E*, dass die „eigene Willenskraft“, und bei den Befragten *mit E* die „E-Zigarette mit Nikotin“ sowohl am meisten genutzt als auch als hilfreichste Methode angegeben wurde.

### Förderliche Faktoren für Rauchstoppversuche

Diejenigen, die mit dem Rauchen von Tabak aufgehört haben (*n* = 3776), wurden danach gefragt, was ihnen für die Motivation zum Rauchstopp geholfen hat (Mehrfachnennungen möglich; Tab. 4).

**Tab. 4:** Hilfen zur Motivation (bezogen auf ehemalige Raucher*innen; Mehrfachnennungen möglich; %)

	Mit E (*n* = 2914)	Ohne E (*n* = 863)	Gesamt (*n* = 3777)
Schlechter Geruch	65	43	60
Gedanken an gesundheitliche Schäden	57	55	57
Gesundheitliche Probleme Atemwege	50	35	46
Gedanken an Geldersparnis	50	33	46
Schlechter Geruchs‑/Geschmackssinn	51	27	45
Andere gesundheitliche Probleme	38	27	35
Info über Regeneration des Körpers	29	27	29
Gedanken an Aufgabe von Abhängigkeit	16	38	21
Vorbildfunktion Kinder	19	19	19
Unterstützung Partner*in	18	17	17
Rauchbedingte Krankheits‑/Todesfälle in Familie etc.	16	11	15
Unterstützung Familie/Freund*innen	11	13	11
Gesundheitsschäden anderer (Passivrauchen)	11	8	10
Rauchverbote	6	7	6
Druck Partner*in	5	6	5
Druck Familie/Freund*innen	3	3	3
Anderer Ort	1	6	2
Ab und zu passiv rauchen können	0,5	1	1
Sonstige	10	23	13

Die Tabelle wurden in Teilen aus [15] übernommen, mit freundlicher Genehmigung © Schulz-Kirchner Verlag GmbH 2024

Die am häufigsten genannte Antwort unter der Gruppe *mit E* ist „schlechter Geruch“ (60 %), gefolgt von „Gedanken an gesundheitliche Schäden“ (57 %) und „schlechter Geruchs‑/Geschmackssinn“ (51 %). Bei der Gruppe *ohne E* ist „Gedanken an gesundheitliche Schäden“ (55 %) die häufigste Antwort, gefolgt von „schlechter Geruch“ (43 %) und „Gedanken an Aufgabe von Abhängigkeit“ (38 %). Es zeigen sich also deutliche Unterschiede zwischen beiden Gruppen, wobei die stärkste Differenz beim Gedanken, die Abhängigkeit aufzugeben, besteht, der für diejenigen mit E‑Zigaretten als Ausstiegshilfe weitaus seltener wichtig ist (Tab. 4).

Frauen nennen deutlich häufiger den „Gedanken an die Aufgabe von Abhängigkeit“ (29 % vs. 19 %; Χ^2^(2, *n* = 3729) = 41,6; *p* < 0,001; OR (*n* = 3718) = 1,81), außerdem die „Unterstützung durch Partner*in“, „Unterstützung durch Familie/Freund*innen“, „sonstige“ sowie einen „anderen Ort“. Im Vergleich nennen Männer öfter den „schlechten Geruchs‑/Geschmackssinn“ (48 % vs. 35 %; Χ^2^(2, *n* = 3729) = 42,5; *p* < 0,001; OR (*n* = 3718) = 0,58), daneben jeweils nur etwas öfter „andere gesundheitliche Probleme“, „Informationen über Regeneration des Körpers“ sowie „schlechten Geruch“.

### Probleme und Hindernisse bei Rauchstoppversuchen

Neben Faktoren, die den Rauchstopp unterstützen, wurden auch Hindernisse in diesem Zusammenhang abgefragt (*n* = 5309; Personen *mit E *und *ohne E*).^11^ Sowohl bei aktuell Rauchenden (*n* = 1533) als auch bei Nichtraucher*innen (*n* = 3776) stellen das größte Hindernis Rituale dar (z. B. „ritualisierte Zigarette zum Kaffee“ (58 % der Raucher*innen; 61 % der Nichtraucher*innen); Tab. 5). Faktoren, die auf manifeste Abhängigkeits- bzw. Entzugssymptome hindeuten, z. B. Reizbarkeit/Unruhe, werden insgesamt deutlich seltener genannt als psychische bzw. psychosoziale Aspekte.

**Tab. 5:** Probleme und Hindernisse, die den Rauchstopp schwerer mach(t)en, nach Rauchstatus (%)

	Aktuell rauchend (*n* = 1555)	Aktuell nicht rauchend (*n* = 3754)	Gesamt (*n* = 5309)	Chi-Quadrat-Test Χ^2^(1) und Signifikanz
Ritualisierte Rauchpausen (Arbeit, Studium etc.)	54	64	61	42,8; *p* < 0,001
Ritualisierte Zigarette zum Kaffee	58	61	60	5,9; *p* < 0,05
Ritualisierte Zigarette zu alkoholischen Getränken	44	55	52	48,1; *p* < 0,001
Rauchende im sozialen Umfeld/Freundeskreis	47	45	45	2,8; n. s.
Andere Situationen mit „Triggerfunktion“ (z. B. Weg zum Bus oder Wartesituation)	36	46	43	40,2; *p* < 0,001
Craving/Verlangen nach Zigaretten/Rauchen	42	44	43	2,0; n. s.
Generelle Tagesstruktur, in die Zigarettenpausen eingebaut waren	33	45	42	70,3; *p* < 0,001
Reizbarkeit, Unruhe	44	32	35	72,0; *p* < 0,001
Erhöhter Appetit und/oder Gewichtszunahme	21	15	17	24,5; *p* < 0,001
Depressive Verstimmungen oder Depressionen	20	12	14	68,5; *p* < 0,001
Konzentrationsschwäche	12	9	10	12,5; *p* < 0,001
Schlafprobleme	13	7	9	43,9; *p* < 0,001
Kopfschmerzen	6	3	4	12,0; *p* < 0,001
Andere psychische Probleme	4	1	2	45,7; *p* < 0,001
Sonstige	5	4	4	6,6; *p* < 0,05

Die Tabelle wurden in Teilen aus [15] übernommen, mit freundlicher Genehmigung © Schulz-Kirchner Verlag GmbH 2024

*n.* *s.* nicht signifikant

Im Gruppenvergleich zwischen der Gruppe *mit E* und *ohne E* fällt auf, dass in der „E-Zigaretten-Gruppe“ häufiger Ritualisierungen oder Trigger genannt werden. Zumindest zum Teil ist dies mit der höheren Nichtraucher*innenquote in der Gruppe erklärbar, aber nicht vollständig.

Im Geschlechtervergleich nennen Frauen mehr als doppelt so häufig „erhöhten Appetit und/oder Gewichtszunahme“ (27 % vs. 13 %; Χ^2^(2, *n* = 5251) = 142,1; *p* < 0,001; OR (*n* = 5237) = 2,47), „depressive Verstimmungen oder Depression“, „Reizbarkeit/Unruhe“ sowie „andere psychische Probleme“. Im Gegensatz hierzu geben Männer hier deutlich öfter die „ritualisierte Zigarette zu alkoholischen Getränken“ (55 % vs. 42 %, Χ^2^(2; *n* = 5251) = 76,6; *p* < 0,001; OR (*n* = 5237) = 0,58), „ritualisierte Rauchpausen“, „andere Situationen mit ‚Triggerfunktion‘“ sowie (nur etwas öfter) „Konzentrationsschwäche“ an.

## Diskussion

Ziel der RauS-Studie war es, zu untersuchen, mit welchen Methoden Rauchstoppversuche unternommen wurden, wie hilfreich diese Methoden aus Sicht der Studienteilnehmer*innen waren und welche Faktoren dabei als förderlich bzw. hinderlich empfunden wurden. Um diese Ziele zu erreichen, wurde eine quantitative Online-Befragung durchgeführt. Teilnehmen konnten Personen, die entweder aktuell Verbrennungszigaretten rauchen oder dies in der Vergangenheit über mindestens 12 Monate hinweg mindestens wöchentlich getan haben. Mit der hier präsentierten Studie stehen erstmals umfangreiche Daten zu förderlichen und hinderlichen Faktoren bei einem Rauchstopp für Deutschland zur Verfügung.

Grundsätzlich zeigt sich, dass die Bereitschaft zum Rauchstopp unter den Befragten hoch ist, was u. a. aus der Art der Rekrutierung resultieren dürfte, da den Teilnehmenden bekannt war, dass Rauchstopp das zentrale Thema der Studie ist. In repräsentativen Stichproben ist die Bereitschaft, das Rauchen einzustellen, für gewöhnlich deutlich geringer [4]. Personen, die den Rauchausstieg geschafft haben, brauchten im Schnitt knapp 4 ernsthafte Versuche. Zur Unterstützung nutzen die Befragten *ohne E* am häufigsten die eigene Willenskraft, was sich mit bisherigen Studien aus Deutschland deckt [4, 5]. Zudem wird diese Methode auch als die hilfreichste bewertet. Auch das deckt sich zumindest mit Studien aus dem internationalen Raum [21]. Dabei handelt es sich im eigentlichen Sinne nicht um eine konkrete „Methode“, um das Rauchen einzustellen, sondern eher um das Vertrauen darauf, den Rauchausstieg ohne Strategie, Hilfsmittel und/oder professionelle Unterstützung bewältigen zu können. International gibt es einzelne Studien, die ergründen, warum die eigene Willenskraft verwendet wird: Dabei wurde neben Misstrauen gegenüber professionellem und als „theoretisch“ empfundenem Wissen u. a. eine weitverbreitete Auffassung ermittelt, nach der die Entscheidung über den Rauchstopp in der eigenen Verantwortung liegen sollte; eigenverantwortliches Aufhören wird dabei als eher kompatibel mit dem jeweiligen Selbstbild und dem Verständnis des Charakters von Nikotinabhängigkeit (nicht als „medizinische Störung“, sondern als persönliches Verhaltensproblem) angesehen [22]. Auch für den deutschsprachigen Raum wäre eine nähere Ergründung der Motive für „eigene Willenskraft“ von Interesse. Einige Resultate der vorliegenden Studie, u. a. zu verwendeten Hilfsmitteln, deuten darauf hin, dass die Auffassung, ein Rauchstopp liege vor allem in der eigenen Verantwortung, auch hierzulande verbreitet sein dürfte.

Von den als evidenzbasiert geltenden Methoden werden am häufigsten Nikotinersatzprodukte genannt, die allerdings eher schlecht bewertet wurden. Daher stellt sich die Frage, ob sich der Fachdiskurs weiterhin derart klar auf diese Methoden konzentrieren sollte – wie überhaupt der Fokus auf die Bekämpfung von pharmakologisch induzierten Entzugserscheinungen infrage zu stellen ist (s. unten). Auffällig ist außerdem, dass „sonstige Methoden“ als sehr hilfreich zur Unterstützung eines Rauchstopps bewertet wurden, wobei sich ein sehr breites Spektrum individueller Strategien und Hilfsmittel zeigte. Dies deutet darauf hin, dass es lohnenswert sein dürfte, in der Rauchentwöhnung derartige individuelle Methoden aktiv zu unterstützen.

Unter den Befragten *mit E* werden E‑Zigaretten mit Nikotin am häufigsten für einen Rauchstoppversuch genutzt, was aufgrund der Charakteristik dieser Teilstichprobe auch zu erwarten war. Überraschend hingegen ist, dass nikotinhaltige E‑Zigaretten auch mit großem Abstand am besten bewertet wurden. Insofern zeigt sich, dass E‑Zigaretten, obwohl sie in Deutschland nicht als offizieller Teil einer gesundheitspolitischen Strategie und der S3-Leitlinien genutzt werden, für eine bestimmte Population eine große Bedeutung zur Schadensminimierung oder zum endgültigen Ausstieg einnehmen.

Bezugnehmend auf die zweite Forschungsfrage nach den hinderlichen und förderlichen Faktoren zeigt sich, dass als förderliche Faktoren in erster Linie (mögliche) gesundheitliche Folgen genannt werden. Als größtes Hemmnis für einen Rauchstoppversuch werden hingegen mit dem Rauchen assoziierte Rituale genannt, während beispielsweise Entzugssymptome eine deutlich weniger bedeutsame Rolle einnehmen. Entzugserscheinungen werden indes von aktuell Rauchenden häufiger genannt als von Personen, die mit dem Rauchen aufgehört haben. Dies zeigt, dass die Angst vor solchen Symptomen ein großes und möglicherweise unbegründetes Hemmnis darstellt, an das leicht mit Präventionsbotschaften angeknüpft werden könnte. In Bezug auf die Hindernisse bietet sich schließlich auch ein näherer Blick auf die Gruppe derer an, die Rauchstoppversuche mittels E‑Zigaretten unternommen haben: In dieser Gruppe werden Rituale und „Trigger“ als besonders wichtig eingeschätzt. Dies könnte ein Hinweis darauf sein, dass es für Personen, für die Ritualisierungen besonders wichtig sind (was auf einen großen Teil der Rauchenden zutrifft), hilfreich ist, eine Ausstiegsmöglichkeit zu nutzen, mit der erlernte Rituale in anderer Form zunächst weitergeführt und ggf. später überwunden werden können. Dies deutet auf das große Potenzial von E‑Produkten als Maßnahme zur Schadensminimierung hin.

Internationale Studien zeigen, dass es durchaus Unterschiede zwischen den Geschlechtern gibt, die sich auf unterschiedlichen Ebenen zeigen [23, 24], und Angehörige von geschlechtlichen Minderheiten möglicherweise spezifische Bedarfe haben [25]. In der „RauS“-Studie bestätigt sich dies bezogen auf einzelne Faktoren: So scheinen für Frauen die subjektive Abhängigkeit sowie wahrgenommene Entzugssymptome, allen voran eine mögliche Gewichtszunahme, bedeutsamer zu sein als für Männer. Sie nutzen auch häufiger diverse konkrete, insbesondere auf Entzugssymptome abzielende Maßnahmen zum Rauchausstieg mit einer großen Ausnahme: Männer sind bei der Nutzung von E‑Zigaretten sehr deutlich überrepräsentiert.

Darüber hinaus zeigt ein Blick in internationale Studien, dass in verschiedenen Gruppen (z. B. Menschen mit psychischen Erkrankungen [26, 27]) spezifische Barrieren und förderliche Faktoren hinzukommen, die in dieser Studie nicht aufgeschlüsselt werden konnten. Zudem gibt es Studien, die darauf hindeuten, dass der sozioökonomische Status Einfluss auf die Motive für einen Rauchstopp sowie auf die Gründe für einen Wiedereinstieg ins Rauchen nimmt [28, 29]. Hier bedarf es weiterer Forschung, um mögliche Unterschiede zu eruieren und einzelne Individuen besser zu unterstützen.

Insgesamt zeigen die Studienergebnisse, dass der bisherige Fokus auf die „Nikotinabhängigkeit“ und die damit einhergehenden Maßnahmen zur Linderung der Entzugssymptome an den Bedarfen von einem erheblichen Teil der Rauchenden vorbeizugehen scheint. Raucher*innen, die vor allem mit dem Rauchen assoziierte Rituale als Hindernis sehen, brauchen eher weitergehende und individuelle Unterstützungsangebote als z. B. Nikotinersatztherapien (was nicht bedeutet, dass Letztere für einen bedeutenden Teil aktuell Rauchender nicht auch eine Hilfe sein können). Daran anschließend deuten die Ergebnisse darauf hin, dass E‑Zigaretten zumindest für einen Teil der Raucher*innen eine schadensminimierende Option sowie eine Unterstützung zum Rauchstopp darstellen können.

### Limitationen

Hinsichtlich der Stichprobe zeigt sich zunächst, dass es zu einer Verzerrung durch die Erwähnung der Studie seitens „Dampfer-Influencer*innen“ kam und damit E‑Zigaretten-Nutzer*innen überproportional häufig vertreten sind. Auch wenn versucht wurde, diesem Umstand durch eine Aufteilung der Stichprobe in 2 Gruppen (*mit E* und *ohne E*) entgegenzuwirken, fällt auf, dass im Vergleich zu Repräsentativerhebungen wie der DEBRA-Studie auch außerhalb der großen Gruppe, die E‑Zigaretten zum Rauchstopp nutzten, auffällig viele Personen Erfahrungen mit E‑Zigaretten haben; möglicherweise ein Anzeichen für einen fortschreitenden Trend in diese Richtung [13]. Dennoch ist denkbar, dass Personen mit E‑Zigaretten-Erfahrung in der Stichprobe auch *generell* überrepräsentiert sind.

Eine weitere Limitation besteht darin, dass es sich generell um eine nichtrepräsentative Erhebung handelt. Die große Deckungsgleichheit zentraler Merkmale der Teilstichprobe „ohne E“ mit Raucher*innen in Deutschland insgesamt spricht allerdings für die Aussagekraft der Ergebnisse.

Auch wenn der Erhebung ein Pretest mit offenen Antwortfeldern vorgeschaltet war, um im Sinne einer qualitativen Forschungslogik neue hinderliche und förderliche Faktoren zu identifizieren, lässt sich kritisch anmerken, dass es weitreichenderer qualitativer Erhebungen mit unterschiedlichen Gruppen (z. B. Frauen, Personen, mit niedrigem sozioökonomischen Status) bedarf, um weitere, möglicherweise relevante Faktoren zu identifizieren.

Es wurden weder Wohnort noch Nationalität oder auch Migrationshintergrund abgefragt; insofern bezieht sich die Stichprobe auf Personen, die der deutschen Sprache mächtig sind. Angesichts der Rekrutierungsformen ist anzunehmen, dass der weit überwiegende Teil der Befragten in Deutschland lebt; Menschen mit weniger ausgeprägten Deutschkenntnissen sind vermutlich unterrepräsentiert.

Im Zusammenhang mit der Anonymität wurden keine Bestrebungen unternommen, um zu verhindern, dass der Fragebogen mehrfach ausgefüllt wurde. Es ist also nicht auszuschließen, dass einzelne Befragte mehr als einmal teilgenommen haben. Dank der Größe der Stichprobe dürfte dies aber kaum ins Gewicht fallen.

Eine generelle Limitation besteht darin, dass Menschen mit geringerem Bildungsniveau weniger bereit sind, an Fragebogenstudien teilzunehmen [27]. Dies könnte ein Grund für den überdurchschnittlichen Bildungsstand in der vorliegenden Stichprobe sein.

## Fazit

Aus den Ergebnissen wird deutlich, dass die wichtigsten Barrieren keineswegs mögliche Entzugssymptome sind, sondern vor allem mit dem Rauchen assoziierte Rituale als Problem empfunden werden. Hier kann angesetzt werden, indem mit Raucher*innen an einem Ersatz für ebendiese Rituale gearbeitet wird. Weiterhin sollte Ausstiegswilligen die gesamte Spanne von Methoden, Ersatzritualen und Hilfsmitteln, inklusive E‑Zigaretten, angeboten werden. Die Fokussierung offizieller Richtlinien auf Maßnahmen zur Bekämpfung der „Nikotinabhängigkeit“ sollte überdacht werden, indem verhaltensbezogene Ansätze sowie Maßnahmen zur Harm Reduction deutlich stärker in den Fokus gerückt werden [30].

## Footnotes

1 E‑Zigaretten wurden im Fragebogen mit folgendem Satz erklärt: „Hier sind Geräte gemeint, die mit Liquids bzw. Kartuschen mit Liquids befüllt werden (oder Einweggeräte mit Liquids).“

2 Tabakerhitzer wurden im Fragebogen mit folgendem Satz erklärt: „Hier sind Geräte gemeint, die mit Tabak-Sticks befüllt werden (z. B. IQOS® oder Glo®).“

3 „Wie häufig haben Sie in Ihrem Leben versucht, mit dem Rauchen aufzuhören? Bitte alle Versuche mitzählen, auch die weniger ernsthaften.“

4 „Wie viele dieser Versuche würden Sie als ernsthafte Versuche bezeichnen? Mit ernsthaftem Versuch meinen wir, dass Sie vorhatten nie wieder zu rauchen.“

5 „Haben bzw. hatten Sie mindestens einmal den Versuch unternommen, das Rauchen deutlich zu reduzieren? Dies bezieht sich auf Versuche der Reduktion ohne die Absicht, gänzlich mit dem Rauchen aufzuhören.“

6 „Ich habe nicht bewusst versucht, meinen Konsum zu reduzieren, sondern mein Konsum wurde unbewusst weniger.“

7 Die entsprechende Frage lautete: „Welche der folgenden Maßnahmen haben Sie jemals unternommen, um Ihren Rauchstopp zu unterstützen?“, versehen mit insgesamt 21 Antwortkategorien plus „sonstige“ mit offenem Antwortfeld (Tab. 2).

8 Der Wortlaut im Fragebogen ist: „Kontrolliertes Rauchen: geplante stufenweise Reduktion des Rauchens bis auf null“.

9 Für die Odds Ratio wurden im Unterschied zu den sonstigen Geschlechtervergleichen die Befragten mit „diversem“ Geschlecht herausgerechnet.

10 „Für wie hilfreich in Bezug auf Ihre(n) Versuch(e), mit dem Rauchen aufzuhören, halten Sie die von Ihnen ausprobierten Maßnahmen?“

11 „Welche Probleme/Hindernisse mach(t)en es Ihnen schwer, mit dem Rauchen aufzuhören?“
